# Accurate Measurement of Strain in Noncontact Surface Deformation Using Subset-Based Digital Image Correlation

**DOI:** 10.1155/2021/4188236

**Published:** 2021-11-24

**Authors:** S. Agnes Shifani, M. S. Godwin Premi

**Affiliations:** ^1^Department of Electronics and Communication Engineering, Sathyabama Institute of Science and Technology, Chennai 602119, Tamil Nadu, India; ^2^School of EEE, Sathyabama Institute of Science and Technology, Chennai 602119, Tamil Nadu, India

## Abstract

The measurement of strain using some contact techniques has some drawbacks like less accuracy and it takes larger computation time for finding each location of subpixels. Thus, a faster noncontact Digital Image Correlation (DIC) mechanism is utilized along with the traditional techniques to measure the strain. The Newton-Raphson (NR) technique is considered to be an accepted mechanism for accurate tracking of different intensity relocation. Generally, the issue regarding the DIC mechanism is its computational cost. In this paper, an interpolation technique is utilized to accomplish a high precision rate and faster image correlation; thereby it reduces the computation time required for finding the matched pixel and viably handles the rehashing relationship process. Hence, the proposed mechanism provides better efficiency along with a reduced number of iterations required for finding the identity. The number of iterations can be reduced using the Sum of Square of Subset Intensity Gradients (SSSIG) method. The evaluation of the projected scheme is tested with different images through various parameters. Finally, the outcome indicates that the projected mechanism takes only a few milliseconds to match the best matching location, whereas the prevailing techniques require 16 seconds for the same operation with the same step size. This demonstrates the effectiveness of the proposed scheme.

## 1. Introduction

DIC is an important and viable optical metrology procedure that can give the full-field displacement and an object's strain distribution. It defeats the restrictions of the contact activity, complex execution, and other different problems. This strategy has gotten one of the most adaptable and well-known procedures in the strain examination field. Generally, DIC [1] depends on the standard of comparing a reference picture and arrangement of altered pictures recorded utilizing a CCD camera. Different correlation functions are characterized to assess the resemblance between the reference and the target pictures. The full-field relocations can be recovered once the greatest correlation coefficient has been recognized. Subsequently, the strain parts can be determined utilizing mathematical differential strategies. Though the standard DIC can accomplish powerful outcomes much of the time, it is hard to acquire exact displacement and strain in discontinuous samples. Different strategies have been created to solve this problem. Finite-element-based strategies that coordinate the limited component strategy into a correlation procedure are well known. The deformation analysis with high accuracy and faster deformation using Digital Image Correlation (DIC) has been greatly demanded in modern times. DIC is an approach utilized for estimating strain and displacement and to think about basic wonders. It takes a look at a movement of photos at different transformation stages and catches the intensity advancement in the region of interest (ROI) and records displacement, length variations, and so forth by constantly focusing on the recorded pictures through coordinate calculations. DIC method is applicable in areas of experimental mechanics as well as in industrial area, especially for both scientific and commercial applications.

The fundamental principle of the most broadly utilized subset-based DIC strategy is matching the similar subsets found in the reference picture and deformed picture to recover the full-field relocations. Strain and relocation are the basic specifications in the valuation of element characteristics like Moiré interferometry; DIC has been generated from previous learning. In photomechanics, the DIC mechanism has been considered as a developing technique by various scientists because of its behaviors like continuity, noncontact activity, whole area estimation, and shrinking of repetitive stage data. Initially, the DIC mechanism was developed in most areas, especially for pressure breakdown and deformation. The term image correlation is used in basic designing functions to a prominent design coordinating approach for the most part utilized in photogrammetric and PC vision to get back the corresponding attention. The framework utilized in [2] could not determine Poisson's module for structural materials; it presents a standard deviation of 57 × 57 and a technical strain of 350 *με*. The key idea after acknowledged and largely used subgroup on DIC methodology is to follow the substitute pictures decided in a real picture through the progression of mutilated images.

The strain is the difference in length in the case of distortion. Several advancements are accessible in an image processing technique to quantify neighborhood displacement and strain like speckle metrology, strain gauge, and Moiré interferometry. Those frameworks have their own limitations like discrete local areas. Moiré interferometry gives strain planning and total dislodging yet it is in fact requesting, restricting, and tedious. In speckle metrology, contrast enhancement is required, since it requires finding the displacement of the material under stress. Some of the strain estimating traditional types of equipment does not provide the exact strain graph. Under these impediments, progression was made to have the previously mentioned anticipated yield. The current progression is the DIC that gives the strain guiding structure of the gross model that is restrictive to mechanical examination. The significance of the current framework allows information around the entire zone development and distortion. References [3, 4] indicate the assessment of metal disfigurement that is utilized in the automobile industry. Here, the exactness depends on the subset size, spot design [5], correlation model, shape work, subpixel insertion plan, and subpixel enlistment calculation [6, 7], and the Newton-Raphson iterative method, semi-Newton procedure, and point-based count have furthermore been proposed to quicken the streamlining circle. In continuous examples, iterative spatial area cross-association procedure is one of the most extensively used subpixel selection estimations, yet the NR method was developed completely by diminishing its computation multifaceted nature. Reference [8] presents another product and the strain yield was better when validating with the existing methodology.

The remainder of this paper is organized as follows: Section 2 investigates some of the related works concerning the Digital Image Correlation scheme.  Section 2.1 provides the problem definition associated with different algorithms while undergoing correlation operation.  Section 2.2 extends a detailed description of the proposed mechanism.  Section 2.3 provides the DIC principle.  Section 2.4 provides the experimental verification, Sections 3 and 4 demonstrates the result and discussion, and Section 5 concludes the paper.

## 2. Related Works

Some of the recent works of literature related to this current research are described below. Some years ago, a developed calculation, Newton-Raphson strategy with halfway algorithmic revision, was utilized; also it was demonstrated that a certain calculation of correlation has been radically diminished in examination together with the subset search calculation [9]. One of the key reasons for its extensive application in numerous sectors of research is its ability to account for relative deformation and rotation of the target subset. In comparison to other approaches, it is also capable of giving the best sub-pixel registration accuracy. NR system needs the correlation work; it ought to be noticed that the underlying theory must be characterized as precisely as conceivable because just along these lines the assembly of the NR approach is ensured. As a rule, this reality gives the likelihood to utilize the techniques which are exceptionally basic from the hypothetical perspective and direct for execution which likewise implies that they are computationally proficient. Yet, the method took large amount of time for computation [10].

As indicated by this, the coarse-fine search calculation is appropriate for such an assignment. Reference [11] shows that, by applying deep learning image correlation, the elastic modulus of nonlinear deformation of images gives the better result with some time consumption. From outset, it figures the simulated correlation value to every focal point in looking through the zone containing a 1-pixel subset. It is essential to limit the searching step to 0.1 pixel or 0.01 pixel, in order to improve its accuracy. From a viable perspective, the estimation of the subset step size relies upon precision that is required in genuine function. Suppose that the subset step size is under 1 subset; the dark layer in alternate-pixel areas should be remade; also, due to this, a specific interpolation plan is needed. This is the most requesting piece of the coarse-fine searching approach during its processing time [12].

The most important estimation method in the area of experimental mechanics is Digital Image Correlation (DIC) because of its flexibility and minimal effort in comparison with other methodologies. Yet, the DIC global image registration executed in MATLAB software could not successfully determine the required complete view transformation with high accuracy, as an image registration kind of “affine” or “similarity” is utilized, based upon the two-dimensional information. Hence, a DIC introduction technique is introduced to assess the surface mishappening of metal sheets utilized in automobile manufacturing. Because of the difficult multifaceted nature, the technique begins with the 3D focuses on recreation [13] to show the four focuses. Consequently, a programmed search is carried out among the close marks to recreate it. Then, the neighborhood DIC is utilized to confirm them as the right marks. The outcome indicates that the method is highly reliable and confidential for experimental cases. But it is computationally complex to begin with 3D focus to depict the other focuses.

In [14], a hybrid genetic algorithm is proposed in which adaptive mechanisms and a simulated annealing mutation process are included in the real-parameter genetic procedure, which is exploited to explore the analogous subset after twisting. To contribute to the precision and dependability of this technique, some fundamental factors are to be measured. The outcomes demonstrate that the out-of-plane move could be incorporated, and a subset with 30 × 30 pixels ought to be suggested. Concerning the searching methodology, it is suggested that the plan factors are separated into three categories, each time just one set is under pursuit, and it takes terms successively. The outcomes demonstrate that this strategy is powerful and constant if its key boundaries are selected properly. The proper selection of boundary is a challenging task.

The coarse-fine search mechanism is found to be accurate and reliable for image correlation if used properly. The coarse-fine searching technique is fit for gradient calculation. Yet, the speed at which it does so has restricted the extensive use. The turn of events and restricted test validation strategy that could decide gradients and displacements utilizing the Newton-Raphson technique [15] for halfway rectifications is introduced. This strategy is exact in deciding particular gradients and displacements when utilizing essentially less CPU time than the present coarse-fine searching technique. The strategy is computationally demanding when utilized for the estimation of displacements and displacement gradients. Another DIC mechanism has been created, which will take into consideration the calculation of displacements and displacement gradients utilizing very less calculation time. The Newton-Raphson procedure is a productive option in contrast to the coarse-fine searching strategy for deciding disfigurements among the digital pictures. The Newton-Raphson strategy utilized minimum computation time compared to the coarse-fine search technique and it determines not only the displacements but also the displacement gradients too. However, a bigger number of iterations are required for fixing the matched region.

The key contribution of our research is summarized as follows: Initially, the original image specimen is taken and the region of interest is plotted. Subsequently, a load is applied to the original image for some period of time to form a deformed image and the region of interest is plotted. The iteration continues for different load conditions. Now, the matching point pixels among the original image and the deformed image are estimated by finding correlation among pixels through an interpolation mechanism. Finally, the amount of strain applied is measured and the computation time for determining the matched pixel location is calculated to determine the efficacy of the system.

### 2.1. Problem Definition

**Figure 1:**
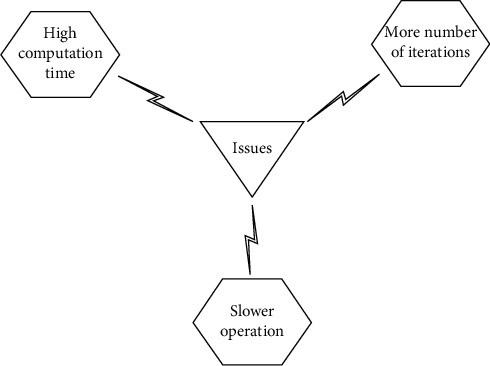
The problem associated with determining matched pixel location.

The main drawback of the iterative spatial gradient is taking many iterations to compare the matching point pixel of the original image and deformed image. Despite the fact that NR algorithm is the most widely used and the most accurate algorithm for subpixel motion estimation, it remains to have extremely huge computational cost.  Figure 1 depicts the problems associated with determining the matched pixel location. The interpolation computation of a pixel point of a specific reference subset is not just acted in all iterations. However, it likewise requires to be completed for a similar pixel point that appeared in neighboring reference subsets. The continuous interpolation computation carried out at each subpixel position takes more time for execution.

### 2.2. Proposed Methodology

**Figure 2:**
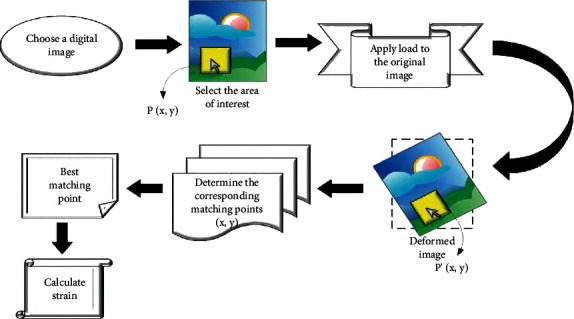
Flow diagram of the proposed method.

Initially, a random digital image is taken and its corresponding target area is chosen. Consequently, a surface recording is performed before the strain. After that, the load is applied so that the image is deformed to some other location after strain. The correlation among the pictures is determined to find the matched location in the deformed image. If the correlation among the pixels is obtained as 1, then that pixel point is said to be matched. Once the corresponding matching points are located, the displacement is measured to calculate the strain in the image. The flow diagram of the proposed strategy is depicted in Figure 2.

Consider the initial subset 1.5 × 1.5 pixel in the reference image and the corresponding matching pair is found in deformed images by applying a new method. Generally, this step is common for all coarse-fine searching method. In every alternate element position of the square subset region, the correlation function should be estimated. The chosen subset is material for looking through the perfect matching pair by effectively inclined searching step in both directions which appears by *x*_step and *y*_step individually.

**Figure 3:**
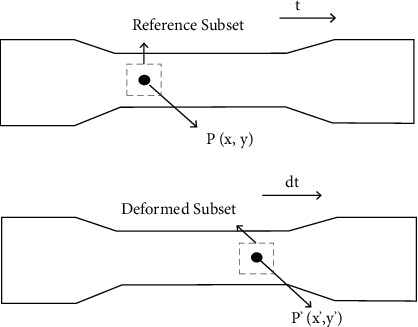
Fundamental deformation concept.

For this reason, fine search strategies exerted a lot of effort to look for and match each subpixel area demonstrating the 0.01 searching step in both directions; then 101 × 101 times were taken for test point. Reference [15] proposed a 0.1-pixel searching step in the two headings; at that point 11 × 11 times were taken for test point. Also, [16] implied 1-pixel searching step in *x* and *y* headings; at that point 2 × 2 times were taken for test point. This reality was the fundamental motivation to build up an improved and better search strategy, wherein computational unpredictability would be altogether diminished. Depending on the accepted searching method, a novel looking through a plan can be characterized through pursuits.  Figure 3 shows the actual specimen along with the deformed image with 0.1x displacement of a pixel.

### 2.3. DIC Principle

Strain estimation is reliant on the relationship of two images acquired by examining the real and deformed pictures. Two subsets are chosen independently from real and deformed examples for calculating the distance. The correlation calculation perceives the neighboring displacements *p* and *q* by considering the above two pictures. The calculations of *p* and *q* could be attained by following the conditions in the following equation [17]:

(1) $$\begin{array}{c} \left\{\begin{array}{c} \frac{\partial I}{\partial x}=0,\\ \frac{\partial I}{\partial y}=0. \end{array}\right. \end{array}$$

Then the displacement area of *p* and *q* would be resolved basically by replacing distinct vector in the above iterating procedure. After the disfigurement augmentation could be portrayed as in the two following equations [17]:

(2) $$\begin{array}{c} x^{\prime }-x=p+\frac{\partial p}{\partial x}\text{ d}x+\frac{\partial p}{\partial y}\text{ d}y. \end{array}$$

(3) $$\begin{array}{c} y^{\prime }-y=q+\frac{\partial q}{\partial x}\text{ d}x+\frac{\partial q}{\partial y}\text{ d}y, \end{array}$$

where (*p*, *q*) indicates the exclusions of the primary mark and (*dx*, *dy*) indicates the contrast locations amidst the primary mark and the neighboring point before deformation. The main deforming angle sections are signified as *∂p*/*∂*x, *∂q*/*∂*x, *∂p*/*∂*y, and *∂q*/*∂*y. Since only the 2D distortion is advised in the conditions in (2) and (3), that needs two dislodging fragments and four displacement slope segments to depict the circumstance of a neighboring point after deformation.

The principal idea behind this strategy is to match the primary cover in the real picture together with a disfigured subset in the image after distortion is depicted in Figure 3. The basic subset is a square along the core pixel in its center. At the point when the area of the target in the deformed picture is found, the development parts of the primary and target area focuses can be settled.

The evaluation of the proposed system is utilized on PC based system which produces spotted pictures that have quite recently used Sum of Square of Subset Intensity Gradients (SSSIG) method; this indicates that the average and variance of picture clamor are the steady characteristics; in this manner, the accurate detection of displacement could be restricted by adjusting SSSIG [17], which could be extended or reduced through modifying the size of the subset. It is furthermore apparent that the accurate displacement detection of DIC can be reasonably enhanced by including two distinctive measures: growing the SSSIG subset and lessening the noise in the picture. The distinction of ∆*p*′ and ∆*q*′ is illustrated using the two following equations:

(4) $$\begin{array}{c} D\left(\Delta p^{\prime }\right)=\frac{\varphi }{\sum \sum {\left(g_{x}\right)}^{2}}.D\left(\sigma^{2}\right), \end{array}$$

(5) $$\begin{array}{c} D\left(\Delta q^{\prime }\right)=\frac{\varphi }{\sum \sum {\left(g_{y}\right)}^{2}}.D\left(\sigma^{2}\right), \end{array}$$

where ∑∑(*g*_*x*_)^2^ and ∑∑(*g*_*y*_)^2^ are the SSSIG in *x* and *y* directions individually,  *D*(*σ*^2^) indicates the image noise variance, and *φ* defines the ratio of the square of cross-correlation coefficient between *g*_*x*_ and *g*_*y*_.

Remarkably, the digital image is portrayed through a restricted intensity count. Hence, one may feel that precision is obliged to another pixel, regardless, at the current reference time; it is possible to find enrollment strategies with exactness better than one pixel. To assess the degree of equivalence in the midst of the underlying and the turned subsets, a particular relationship measure should be described at first. The underlying strategy is the utilization of estimation along with one-pixel accuracy. This can be acquired by an essential discovering plan inside the curved picture or with a further evolved strategy. In [18], the manufacturers developed a speedy recursive arrangement to experimentally decrease the computational multifaceted nature of the ordinary DIC strategy with one-pixel accuracy.

**Figure 4:**
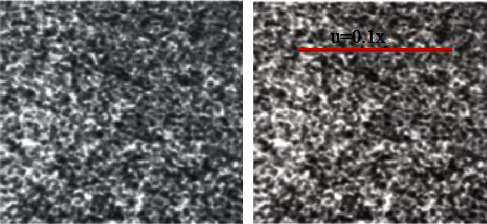
The surface of the specimen with displacement.

Figure 4 represents the surface of the specimen with some displacement. The idea under the proposed calculation is obviously shown. It is apparent that the plan should be processed on 2.56 occasions; this is greatly more improved compared to the traditional method. As per hypothetical certainty, the quantities of cycles that should be determined for each example point at various searching steps.

At first, the square subset is advised to 1.5 × 1.5 pixels focused in the area of (*x*, *y*). The square subset pixels focused at the area inclined by the proposed algorithm are partitioned into four equal subsets focused at subpixel areas. Looking for the coordinating point at step size of 0.75 pixels is presented. For every one of them, the SSSIG is also determined as the best match is indicated as (*x*_1_, *y*_1_). In subsequent advance, the step size of 0.75 is decreased to 0.375 pixels and then the best coordinating point at another area (*x*_2_, *y*_2_) is analyzed. This strategy is rehashed until the estimation of the searching step is adequately little contrasted with some predefined limit. On the off chance that the searching step is thought to be portioned by *n* searching step for the two bearings, at that point, the square subset pixels focused at (*x*_*n*−1_, *y*_*n*−1_) display the best match, which is indicated by (*x*_*n*_, *y*_*n*_).

**Figure 5:**
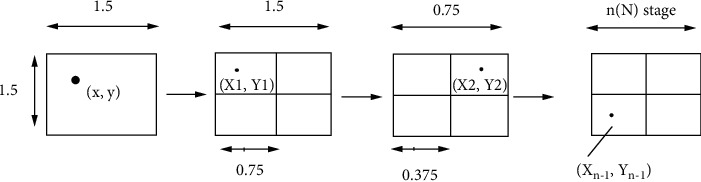
Pictorial representation of the proposed algorithm.

Figure 5 specifies the location of selected pixel point at varying step size. By and large, the places of the initial point and the close through location after transformation are no more situated in subset purposes of the image appropriated after distortion; then there are no dark range qualities for these focuses. Consequently, to find precisely the situation of subpixel, the cross-correlation and some sort of subpixel interpolation calculation [19] are important to recover their dim level qualities with the end goal that the force example of the region could be acquired.

Our work promotes a bilinear interpolation strategy to assess the power level dim slope in the subpixel area. A bilinear interpolation mechanism adds the pixels by identifying sharper edges than the traditional bilinear procedure in the recreated pictures but then enhanced independent qualities. Bilinear interpolation is a resampling strategy that utilizes the distance weighted average of the four closest pixels to assess another pixel. The weights are applied on the basis of the distance of the four nearest pixels leveling the output frame. A bilinear image interpolation examines the three pixels which improve or reduce the real picture pixels in both vertical and horizontal paths, respectively. The intensities at subpixel positions should be recreated and, to achieve this, the subpixel interpolation process should be utilized.

In bilinear interpolation, the intensity level worth *I* (*u*, *v*) [20] at a location situated amid four close pixel focuses is acquired as

(6) $$\begin{array}{c} I\left(u,v\right)=s_{1}u+s_{2}v+s_{3}uv+s_{4}, \end{array}$$

where *s*_*i*_  are constants acquired from the location and grey level estimations of four close intensity focuses. To speak to the correlation of the two above-mentioned regions, a least-squares correlation coefficient or cross-correlation coefficient is regularly utilized. For effortlessness, this least-squares correlation coefficient *γ* is used in this study and characterized using the following equation:

(7) $$\begin{array}{c} \gamma =\sum_{m,n=-R/2}^{R/2}{\left[P_{1}\left(u_{m},v_{n}\right)-P_{2}\left(u_{m}^{\prime },v_{n}^{\prime }\right)+q\right]}^{2}, \end{array}$$

where *P*_1_(*u*_*m*_, *v*_*n*_) and *P*_2_(*u*_*m*_′, *v*_*n*_′) represent the intensities of the location in the regions from the reference image and the deformed image, individually. *R* × *R* means the size of the subset.

**Figure 6:**
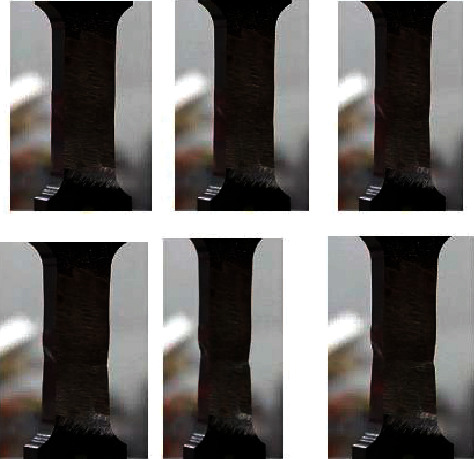
Specimen with different load inputs.

As displayed in the past work [21], subpixel interpolation strategy that ascertains intensity at any alternate-pixel area is a very tedious cause of it requiring some investment at every cycle venture of the fine searching approach. What is more, also for this situation, every pixel in the subset under consideration will be interpolated 4 × *n* times. All the more correctly, if the searching step is thought to be the subset size which is similarly separated by four for the two bearings, at that point, the proposed methodology will be executed 2.56 times [22] at each example point. Here, it demonstrates the opportunity to cover with the nearby subsets because the pixel location inside one initial subset may likewise show up in its nearby original subset as shown in Figure 6. Reference [23] implies the dull interpolation ought to stay away from on a similar pixel location. As an initial subset of (2R + 1) × (2R + 1) pixels measurement and a grid step of ∆*L* pixels, every pixel in this region is then likewise utilized in the nearby [floor((2*R*+1/Δ*L*)+1)]^2^ reference subsets. On the off chance that the accepted pixel location is uprooted to an alternate-pixel area, this pixel point will be added around *n*/*N*[floor((2*R*+1/Δ*L*)+1)]^2^ times utilizing the regular method, where *n* represents the initial searching step size and *N* is 2, 4, 8,…. Clearly, larger step size and reduced grid step will further diminish the calculation time.

### 2.4. Experimental Verification

To confirm the full-field displacement counts utilizing the fixed-point usage of the spatial gradient strategy, a uniaxial pressure exploration is directed on a rectangular aluminum sample. Uniaxial pressure is applied to the sample utilizing a Universal Testing Machine (UTM). The moveable grasp is dislodged by a controlled sum, as well as a webcam associated with a workstation phone used to catch a picture of a locale of enthusiasm on the sample's surface between the holds. The pictures put away in a workstation phone moved to the inserted microchip module, where the subpixel DIC calculation is utilized to ascertain the full-field dislodging.

The arrangement begins with the sample in the holds of the UTM, yet with no load applied. In the stacking stage at each stage, the load is applied to move the movable hold at a pace of 0.005 inches/second for an aggregate of 60 seconds; along these lines, each stacking stage extends the example by 0.01 inches. In the holding period of each stage, the grasp is held in position for 60 seconds, with the goal that a picture of the sample under static burden might be taken. An undisfigured picture, arrange 0, is taken before the examination begins. Figure 6 shows the various phases of the sample with different burden disfigurement.

## 3. Results

The proposed mechanism is implemented in MATLAB software. The proposed method aims to calculate the strain applied in the image. At first, the digital image is taken randomly and its corresponding target area is selected. Then, a surface recording is accomplished before applying the load. After applying the load, the image is distorted to some other position due to the strain. The correlation among the pixels is determined to find the matched position in the deformed picture. If the correlation value is unity, then the region is said to be matched. Once the corresponding matching points are located, the displacement is measured to calculate the strain in the image. The strain is calculated by computing the variation in distance length from the ROI of original picture to the deformed picture.

Strain estimation is assessed utilizing test pictures by applying a newly proposed technique; additionally testing examples at that point was introduced into a loading casing and dot examples were procured at different stacking conditions. To assess the exactness and computational expense of the proposed strategy, a rectangular region in the first picture is picked to be the ROI. The built application is used to validate both the proposed and standard fine searching strategies.  Table 1 formulates the computation time for various searching steps for actual and proposed methods.

**Figure 7:**
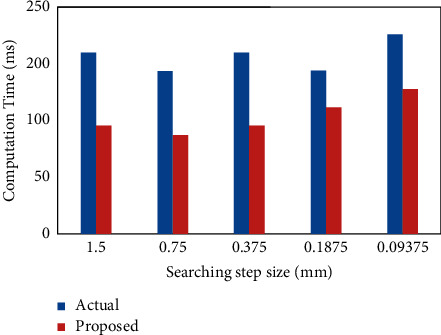
Comparison of computation time versus different searching step between the conventional method and the proposed method.

Figure 7 looks at the calculation speed of the regular plan in comparison to the new plan at changed searching step size, running from 1.5 to 0.375 subset to a fixed subset of 41 × 41 pixel size. As is shown, the calculation rate of the ordinary methodology starts to increment quickly with the finding steps reductions. Consider that the estimation of the subset step size is thought to be 0.375. The traditional method demands more noteworthy time for all example locations to locate the exact match. Then, again, the new methodology just demands a couple of moment's seconds at the equivalent searching steps. From this reality, it is obvious that the calculation time is amazingly decreased.

Table 2 represents the comparison result of the computation time of the conventional approach and the proposed approach. The proposed calculations were tested to correlate discrete picture subsets from the initial and after loaded image and this outcome is contrasted as well as regular outcome Newton-Raphson strategy and FAS calculation [23].

**Figure 8:**
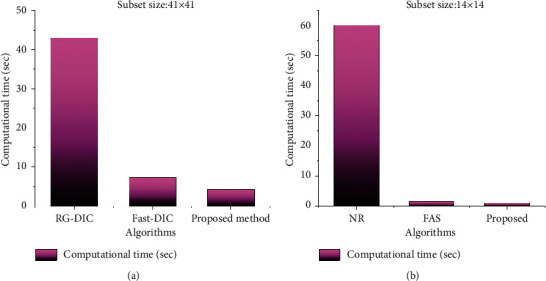
Comparison of computational time for the proposed method and existing methodologies. (a) Subset size: 41 × 41; (b) subset size: 14 × 14.

The performance comparison of the proposed technique and existing techniques for computation time is depicted in Figure 8. From the figure, it is clear that the proposed method takes very less amount of time compared to the prevailing techniques like Fast-DIC and RG-DIC [23] for computing the correlation among the original picture and the deformed picture.

**Figure 9:**
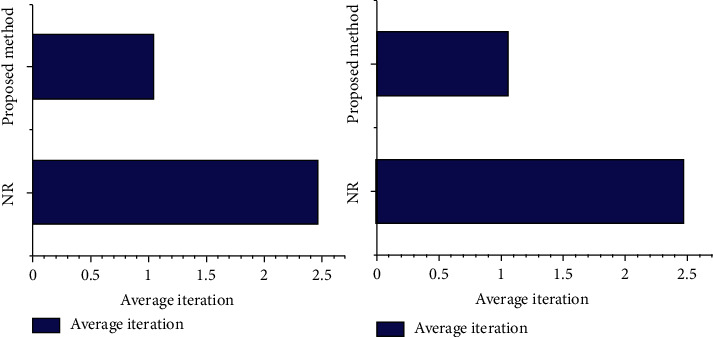
Comparison of average iteration for the proposed method and the existing methods.

The evaluation of average number of iterations required for computation in both the proposed algorithm and existing algorithms is framed in Table 3. It indicates that the NR algorithm took an average iteration in the order of 2.463 points/second, whereas the proposed mechanism took an average iteration in the order of 1.042 points/second for a subset size of 41 × 41, and the NR algorithm took an average iteration of 2.472 points/second, whereas the proposed mechanism took 1.057 points/second for a subset size of 21 × 21. The pictorial representation of the average iteration comparison for the proposed mechanism and existing mechanisms is represented in Figure 9. From the figure, it is clear that the proposed scheme performs better than the prevailing methods.

## 4. Discussion

Image subset-based calculation for image correlation proposed in the present exploration is a framework handling picture pixel coincidence with spot size. The size of the subset determined the region which depends on the varieties of dim levels. The choice of subset territory is steady through the connection strategy; chiefly two imperative terms which will involve the strain exactness must be esteemed. From the beginning, the amount of geometric bending inside the subset region in the disfigured picture must be little enough. Next, the extent and the period of force changes inside the subset region must be extraordinary to give the required strain distribution. So, the choice of the strain of the indicated subset zone needs to settle the thought of more than two components.

On this premise, this exploration was settled on an experimentation concept. In request to lessen the computational speed, an assortment of uses in picture preparation is performed. From our result, implementing new calculation dependent on subimage area division, it is demonstrated that the computational speed is diminished than the conventional technique recently proposed in this work. In the function of Fourier transform on correlation, phase types are used to detect an impulse about pixel variations. In the tensile test, the examination consequence of strain of the traditional approaches and the proposed approach was discussed. From that moment, deviation was found during the count of strain estimation. That was because of little bends and inappropriate dot size.

While comparing the performance of the existing and proposed approaches, it is clear that the proposed mechanism requires a reduced number of iterations for determining the matched pixel, whereas the prevailing techniques take more iterations; that is, the average numbers of iterations required for the proposed method are 1.042 and 1.057, whereas the average numbers of iterations required for the existing NR method are 2.463 and 2.472, respectively, for step sizes of 41 × 41 and 14 × 14. On analyzing the computation time required for finding the matched area in the image, the projected methodology takes less amount of time compared to the existing RG-DIC and Fast-DIC techniques; that is, the proposed method takes 4.34 s for a subset size of 41 × 41, whereas the prevailing RG-DIC and Fast-DIC methods take 42.97 s and 7.35 s, respectively; for a subset size of 14 × 14, the proposed method takes 1.01 s, whereas the prevailing NR and FAS methods take 60 s and 1.5 s, respectively. Thus, the projected scheme is much faster in determining the image correlation and this proves the worth of the system.

## 5. Conclusion

An efficient DIC technique based enhanced subset is proposed in this work for rapid and precise strain estimation. Likewise, it viably diminished rehashed correlation ventures during execution. Subsequently, the tedious number displacement required in the traditional DIC technique is altogether stayed away from in the proposed DIC strategy. Additionally, with the interpolation coefficient of every interpolation obstruct in the deformed picture, excess subpixel interpolation figuring can be radically diminished in the proposed DIC technique. In conclusion, the methodology is a lot quicker than the current ones if a similar precision is expected and in this manner has a favorable position over the past plan in perspective on computing time. Specifically, it is appropriate to be utilized as an ongoing preparing device with specific speed prerequisites. It reasons that a mix of DIC with iterative strategies and Fourier transform can bring about an increasingly precise progressively mechanized and progressively keen estimation strategy for estimating the whole area displacements of huge displacements and finite disfigurement.

## Figures and Tables

**Table 1:** Computation time for various searching steps for actual and proposed methods.

Computation time (ms)		
Searching step size	Actual method	Proposed method
1.5	200	120
0.75	180	110
0.375	200	120
0.1875	180	140
0.09375	220	160

**Table 2:** Computation time between different algorithms.

Subset size	Algorithm	Computation time (sec)
41 × 41	RG-DIC	42.97
	Fast-DIC	7.35
	Proposed method	4.34

14 × 14	NR	60
	FAS	1.5
	Proposed method	1.01

**Table 3:** Comparison of average iteration among various algorithms.

Subset size	Algorithm	Average iteration
41 × 41	NR	2.463
	Proposed method	1.042

21 × 21	NR	2.472
	Proposed method	1.057

## Data Availability

The data used to support the findings of this study are included within the article.
